# Supplementary material for: Identification of Freezing-Responsive microRNAs and Their Targets in Chinese Jujube by Small RNA and Degradome Sequencing
Source: Int J Mol Sci. 2026 Sep 4;27(17):7912. doi: 10.3390/ijms27177912 (PMC13566890; doi:10.3390/ijms27177912)
Supplement: Supplementary file 1 [file ijms-27-07912-s001.zip › Supplementary Material S2/novel_miR_5.pdf]

Provisional ID	:	novel_miR_5
Score total	:	590.3
Score for star read(s)	:	3.9
Score for read counts	:	583.9
Score for mfe	:	0.9
Score for randfold	:	1.6
Score for cons. seed	:	
Total read count	:	1157
Mature read count	:	609
Loop read count	:	546
Star read count	:	2

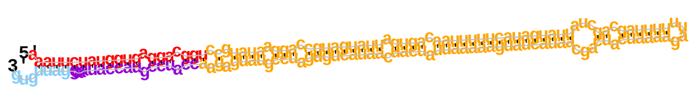

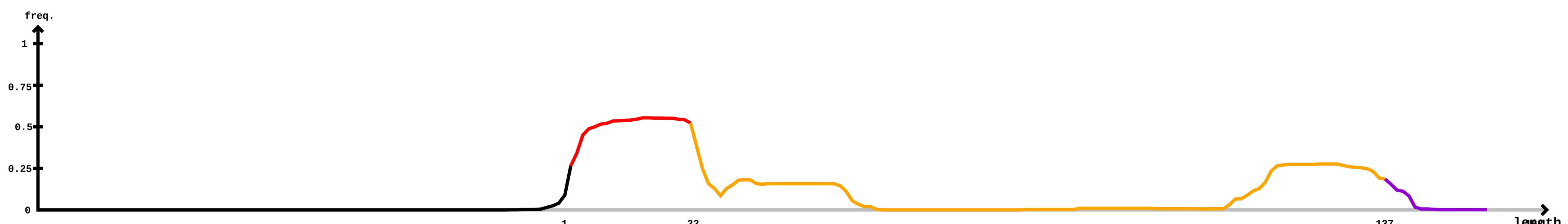

Mature Star

```
5'- ucaccuaaauuauuuuggacggcaagugauauaaaauugacacuuuuugauaaguuaguuuauauauauauauauauauauauacagaaauucuauggugaggacgguccguauaaggaccguaguauuagugacaauuuuucauaguauuaucgacgauuuuuuagaaaaucaucagcaauacuaugaaaaauuaucaucaauacugugauccguaugagaaccauccguaccauaaacggauugugua -3'  obs
    ucaccuaaauuauuuuggacggcaagugauauaaaauugacacuuuuugauaaguuaguuuauauauauauauauauauauauacagaaauucuauggugaggacgguccguauaaggaccguaguauuagugacaauuuuucauaguauuaucgacgauuuuuuagaaaaucaucagcaauacuaugaaaaauuaucaucaauacugugauccguaugagaaccauccguaccauaaacggauuguguua      exp
    .....(((((((.((((..(((.(((((.((......)).))))).))).)))).)))))))(((((((....)))))))((((..(((((((((((.(((.(((.(.((((.(((.((((((((.((((.(((((((((((((((...((.((((((....)))))).))...))))))))))))))).)))).))))))))).))).)))).).))).))).)))))))...))))).))))
```

read	reads	mm	sample
auauauacUgaaauucuaugg	1	1	LT-1
auauacagaaauucuaugguga	2	0	JCK-2
auacagaaauucuauggugaggac	1	0	JCK-1
uacUgaaauucuauggugaggac	2	1	LT-3
acNgaaauucuauggugagg	1	1	LT-2
acagaaauucuauggugaggac	2	0	LT-1
acagaaauucuaugAugaggacgg	2	1	JCK-3
acagaaauucuaugAugaggacgg	1	1	JCK-1
acagaaauucuaugAugaggacgg	4	1	JCK-2
acUgaaauucuauggugaggacgg	2	1	JCK-2
acUgaaauucuauggugaggacgg	1	1	JCK-1
cagaaauuUuauggugaggac	1	1	JCK-3
cagaaauuUuauggugaggacg	1	1	LT-1
caAaaauucuauggugaggacggu	1	1	JCK-1
cagaaauucuauggugaggacggu	1	0	JCK-3
cUgaaauucuauggugaggacggu	2	1	JCK-2
caAaaauucuauggugaggacggu	2	1	JCK-3
cUgaaauucuauggugaggacggu	1	1	JCK-3
cagaaauucuauggugaggacggu	3	0	JCK-2
agaaauucuauggugaggacggu	1	0	JCK-1
agaaauucuauggugaggacggu	1	0	JCK-3
agaaauucuaugAugaggacggu	5	1	JT-1
agaaauucuauggugaggacggu	1	0	JCK-2
agaaauucuauggugaggacgAuc	3	1	JCK-1
agaaauucuGuggugaggacgguc	2	1	JCK-2
agaaauucuGuggugaggacgguc	1	1	JCK-3
agaaauucuaugAugaggacgguc	1	1	JCK-3
agaaauucuauggugaggacgAuc	2	1	JCK-3
agaaauucuauggugaggacgAuc	3	1	JCK-2
gaaauucuauggugaggacg	1	0	JCK-2
gaaauucuauggugaggacg	1	0	JCK-1
gaUauucuauggugaggacgg	2	1	JCK-1
gaaauucuauggugaggacgg	1	0	JCK-2

Mature (red) **Star** (purple)

```
ucaccuaaauuauuuuggacggcaagugauauaaaauugacacuuuuugauaaguuaguuuauauauauauauauauauauauacagaaauucuauggugaggacgguccguauaaggaccguaguauuagugacaauuuuucauaguauuaucgacgauuuuuuagaaaaucaucagcaauacuaugaaaaauuaucaucaauacugugauccguaugagaaccauccguaccauaaacggauugugua
......................................................................................gaaauucuauggugaggacgg...............................................................................................................................................   1   0   JCK-3
......................................................................................gaUauucuauggugaggacgg...............................................................................................................................................   2   1   JCK-2
......................................................................................gaaauuGuauggugaggacgg...............................................................................................................................................   1   1   JCK-3
......................................................................................gaaauuGuauggugaggacgg...............................................................................................................................................   1   1   JCK-1
......................................................................................gaaauucuauggugaggacggu..............................................................................................................................................   6   0   JCK-2
......................................................................................gaaauucuaugUugaggacggu..............................................................................................................................................   2   1   JCK-2
......................................................................................Caaauucuauggugaggacggu..............................................................................................................................................   3   1   JCK-3
......................................................................................gaaauucuaugUugaggacggu..............................................................................................................................................   1   1   JCK-3
......................................................................................gaaauuGuauggugaggacggu..............................................................................................................................................   1   1   JCK-3
......................................................................................Caaauucuauggugaggacggu..............................................................................................................................................   1   1   JCK-1
......................................................................................Caaauucuauggugaggacggu..............................................................................................................................................   2   1   JCK-2
......................................................................................gaaauucuauggugaggacggu..............................................................................................................................................   5   0   JCK-3
......................................................................................gaaauucuauggugaggacggu..............................................................................................................................................   2   0   JCK-1
......................................................................................gaaauucuauggugaggacgguc.............................................................................................................................................   1   0   LT-3
......................................................................................gaaauucuaugCugaggacgguc.............................................................................................................................................   1   1   JCK-2
......................................................................................gaaauucuaugCugaggacgguc.............................................................................................................................................   2   1   JCK-3
......................................................................................gaaauucuauggugaggacggucc............................................................................................................................................   3   0   JCK-3
......................................................................................gaaauucuauggugaggacUgucc............................................................................................................................................   1   1   JCK-2
......................................................................................gaaauucuauUgugaggacggucc............................................................................................................................................   2   1   JCK-3
......................................................................................gaaauucuauggugaggacgguGc............................................................................................................................................   1   1   LT-3
......................................................................................gaaauucuauggugaUgacggucc............................................................................................................................................   2   1   LT-1
......................................................................................gaaauucuauUgugaggacggucc............................................................................................................................................   2   1   JCK-2
......................................................................................gaaauucuauggugaggacUgucc............................................................................................................................................   1   1   JCK-1
......................................................................................gaaauucuauggugaggacggucc............................................................................................................................................   8   0   JCK-2
......................................................................................gaaauucuauggugaggacggucc............................................................................................................................................   2   0   LT-1
.......................................................................................aaauucuauggugaggac.................................................................................................................................................   2   0   LT-3
.......................................................................................aaauucuauggugaggacgg...............................................................................................................................................   1   0   JCK-1
.......................................................................................Caauucuauggugaggacggu..............................................................................................................................................   1   1   JT-1
.......................................................................................aaauucuauggugaggacggu..............................................................................................................................................   43   0   JCK-3
.......................................................................................aaauucuaugUugaggacggu..............................................................................................................................................   1   1   LT-3
.......................................................................................aaauucuauggugaggacggu..............................................................................................................................................   69   0   JCK-2
.......................................................................................aaauucuauggugaggacggu..............................................................................................................................................   9   0   LT-1
.......................................................................................aaauucuauggugaggacggu..............................................................................................................................................   15   0   JT-1
.......................................................................................aaauucuaugUugaggacggu..............................................................................................................................................   1   1   JCK-3
.......................................................................................aaauucuaugUugaggacggu..............................................................................................................................................   8   1   JCK-2
.......................................................................................aaauucuaugUugaggacggu..............................................................................................................................................   7   1   JCK-1
.......................................................................................aaauucuauggugaggacggu..............................................................................................................................................   2   0   LCK-3
.......................................................................................aaCuucuauggugaggacggu..............................................................................................................................................   1   1   JCK-2
.......................................................................................aaauucuauggugaggacggu..............................................................................................................................................   37   0   JCK-1
.......................................................................................aaauucuauggugaggacggu..............................................................................................................................................   5   0   LT-3
.......................................................................................aaauucuauggugaggacgguc.............................................................................................................................................   2   0   JCK-2
.......................................................................................aaauucuauggugaggacgguc.............................................................................................................................................   1   0   JCK-3
.......................................................................................aaauucuaugCugaggacgguc.............................................................................................................................................   2   1   JCK-3
.......................................................................................aaauucuauggugaggacgguccg...........................................................................................................................................   2   0   LT-3
.......................................................................................aaauucuauggugaggacgguccg...........................................................................................................................................   6   0   JCK-2
.......................................................................................aaauucuauggugaggacgguccg...........................................................................................................................................   3   0   JCK-3
.......................................................................................aaauucuauggugaggacgguccg...........................................................................................................................................   2   0   JCK-1
.......................................................................................aaauucuauggugaggacgguccAu..........................................................................................................................................   1   1   LCK-3
........................................................................................aauucuauggugaggacgg...............................................................................................................................................   1   0   JCK-2
........................................................................................aauucuauggugaggacggu..............................................................................................................................................   10   0   JCK-3
........................................................................................aauucuauggugaggacggu..............................................................................................................................................   4   0   JCK-1
........................................................................................aauucuauggugaggacggu..............................................................................................................................................   3   0   LT-1
........................................................................................aCuucuauggugaggacggu..............................................................................................................................................   1   1   LT-2
........................................................................................aauCcuauggugaggacggu..............................................................................................................................................   1   1   JCK-3
........................................................................................aauucuauggugaggacggu..............................................................................................................................................   3   0   LT-3
........................................................................................aauucuauggugaggacggu..............................................................................................................................................   15   0   JCK-2
........................................................................................aauucuauggugaggacggu..............................................................................................................................................   7   0   LT-2
........................................................................................aauucuauggugaggacgguc.............................................................................................................................................   6   0   JCK-3
........................................................................................aauuGuauggugaggacgguc.............................................................................................................................................   1   1   LT-3
........................................................................................aauucuaugCugaggacgguc.............................................................................................................................................   1   1   JCK-3
........................................................................................aauucuauggugaggacgguc.............................................................................................................................................   4   0   JCK-1
........................................................................................aauucuauggugaggacgguc.............................................................................................................................................   5   0   JCK-2
........................................................................................aauucuaugguNaggacggucc............................................................................................................................................   1   1   JT-2
........................................................................................aauucuauggugaggacggucc............................................................................................................................................   5   0   JT-3
........................................................................................aauucuauggugaggacggucc............................................................................................................................................   3   0   LT-1
........................................................................................aauucuauggugaggacggucc............................................................................................................................................   2   0   JT-2
........................................................................................aauucuauggugaggacggucc............................................................................................................................................   2   0   JCK-2
........................................................................................aauucuauggugaggacggucc............................................................................................................................................   2   0   LT-3
........................................................................................aauucuauggugaggacgguccg...........................................................................................................................................   2   0   LT-2
........................................................................................aauucuauggugaggacgguccg...........................................................................................................................................   2   0   JCK-3
```

```
                                                                                        Mature                                                                                                                                 Star
ucaccuaaauuauuuuggacggcaagugauauaaaauugacacuuuuugauaaguuaguuuauauauauauauauauauauauacagaaauucuauggugaggacgguccguauaaggaccguaguauuagugacaauuuuucauaguauuaucgacgauuuuuuagaaaaucaucagcaauacuaugaaaaauuaucaucaauacugugauccguaugagaaccauccguaccauaaacggauugugua
........................................................................................aauucuauggugaggacgguccg...........................................................................................................................................   1   0   LT-1
........................................................................................aauucuaugUugaggacgguccg...........................................................................................................................................   1   1   LT-2
........................................................................................aauucuauggugaggacgguccg...........................................................................................................................................   1   0   LCK-3
........................................................................................aauuGuauggugaggacgguccg...........................................................................................................................................   3   1   JCK-2
........................................................................................aauucuauggugaggacgguccAu..........................................................................................................................................   1   1   LT-2
........................................................................................aauucuauggugaggacgguccgu..........................................................................................................................................   1   0   LT-3
........................................................................................aauucuauggugaggacgguccAu..........................................................................................................................................   1   1   JCK-1
........................................................................................aauucuauggugaggacgguccUu..........................................................................................................................................   2   1   JCK-1
........................................................................................aauucuauggugaggacgguccAu..........................................................................................................................................   1   1   LT-3
.........................................................................................auucuauggugaggacgg...............................................................................................................................................   1   0   JCK-2
.........................................................................................auucuauggugaggacgg...............................................................................................................................................   1   0   JCK-1
.........................................................................................auucuauggugaggacgg...............................................................................................................................................   1   0   JCK-3
.........................................................................................auucuauggugaggacgg...............................................................................................................................................   1   0   LT-3
.........................................................................................auucuauggugCggacgg...............................................................................................................................................   1   1   JCK-2
.........................................................................................auucuauggugaggacggu..............................................................................................................................................   3   0   LT-3
.........................................................................................auucuauggCgaggacggu..............................................................................................................................................   1   1   JCK-2
.........................................................................................auucuaugCugaggacggu..............................................................................................................................................   1   1   JCK-3
.........................................................................................auucuauggugaggacggu..............................................................................................................................................   1   0   LT-2
.........................................................................................auucuauggugaggacggC..............................................................................................................................................   2   1   JCK-3
.........................................................................................auucuauggugaggacggu..............................................................................................................................................   1   0   JCK-3
.........................................................................................auucuauggugaggacggu..............................................................................................................................................   3   0   JCK-2
.........................................................................................auucuauggugaggacggu..............................................................................................................................................   2   0   JCK-1
.........................................................................................auucuauggugaggacgguc.............................................................................................................................................   2   0   JCK-2
.........................................................................................auucuauggugaUgacgguc.............................................................................................................................................   1   1   LT-1
.........................................................................................auucuauggugaggacgguc.............................................................................................................................................   1   0   JCK-1
.........................................................................................auucuauggugaUgacggucc............................................................................................................................................   1   1   LT-1
.........................................................................................auucuauggugaggacggucc............................................................................................................................................   4   0   LCK-3
.........................................................................................auucuauggugaggacgguGc............................................................................................................................................   2   1   JCK-3
.........................................................................................auucuauggugaggacggucc............................................................................................................................................   7   0   LT-1
.........................................................................................auucuauggugaggacggucc............................................................................................................................................   4   0   JCK-3
.........................................................................................auucuauggugaggacggucc............................................................................................................................................   7   0   LT-2
.........................................................................................auucuauggugaggacggucc............................................................................................................................................   5   0   JCK-1
.........................................................................................auucuauggugaggacggucc............................................................................................................................................   10  0   LT-3
.........................................................................................auucuauggugaggacggucc............................................................................................................................................   7   0   JCK-2
.........................................................................................auuGuauggugaggacggucc............................................................................................................................................   1   1   JCK-3
.........................................................................................auucuauggugaggacgguccg...........................................................................................................................................   5   0   JCK-2
.........................................................................................auucuauggugaggacgguccg...........................................................................................................................................   1   0   JCK-1
.........................................................................................auucuauggugaggacgguccg...........................................................................................................................................   2   0   LT-3
.........................................................................................auucuauggugaggaAgguccg...........................................................................................................................................   1   1   JCK-1
.........................................................................................auucuauggugaggacgguccg...........................................................................................................................................   3   0   LT-1
.........................................................................................auucuauggugaggacgguccU...........................................................................................................................................   1   1   LT-1
.........................................................................................auucuaugCugaggacgguccg...........................................................................................................................................   1   1   LT-3
.........................................................................................auucuauggugaggacgguccg...........................................................................................................................................   1   0   LT-2
.........................................................................................auucuauggugaggacgguccgu..........................................................................................................................................   7   0   JCK-2
.........................................................................................auucuauggugaggacgguccgu..........................................................................................................................................   3   0   LT-2
.........................................................................................auucuauggugaggacgguccAu..........................................................................................................................................   2   1   LT-3
.........................................................................................auucuauggugaggacgguccgu..........................................................................................................................................   8   0   LT-3
.........................................................................................auucuauggugaggacgguccAu..........................................................................................................................................   1   1   LT-1
.........................................................................................auucuauggugaggacgguccAu..........................................................................................................................................   2   1   JCK-2
.........................................................................................auucuauggugaggacgguccAu..........................................................................................................................................   1   1   JCK-1
.........................................................................................auucuauggugaggacggAccgu..........................................................................................................................................   3   1   LT-3
.........................................................................................auucuauggugaggacgguccgu..........................................................................................................................................   11  0   LCK-3
.........................................................................................auucuauggugaggacgguccgu..........................................................................................................................................   7   0   LT-1
.........................................................................................auucuauggugaggacgguccgua.........................................................................................................................................   2   0   JCK-2
.........................................................................................auucuauggugaggacgguccgua.........................................................................................................................................   1   0   LT-3
.........................................................................................auucuauggugaggacgguccgua.........................................................................................................................................   1   0   JCK-1
..........................................................................................uucuauggugaggacggu..............................................................................................................................................   6   0   LT-2
..........................................................................................uucuauggugaggacggu..............................................................................................................................................   2   0   JCK-3
..........................................................................................uucuauggugUggacggu..............................................................................................................................................   1   1   LT-1
..........................................................................................uucuauggugaggacggu..............................................................................................................................................   1   0   LT-3
..........................................................................................uucuauggugaggacggu..............................................................................................................................................   1   0   JCK-1
..........................................................................................uucuauggugaggacggu..............................................................................................................................................   1   0   JCK-2
..........................................................................................uucuauggugaUgacggu..............................................................................................................................................   1   1   LT-3
..........................................................................................uucuauggugaUgacggucc............................................................................................................................................   1   1   LT-1
..........................................................................................uucuauggugaUgacggucc............................................................................................................................................   1   1   LT-2
..........................................................................................uucuauggugaggacggucc............................................................................................................................................   1   0   LT-1
..........................................................................................uucuaugCugaggacggucc............................................................................................................................................   1   1   JCK-3
..........................................................................................uucuaugCugaggacggucc............................................................................................................................................   1   1   JCK-1
..........................................................................................uucuauggugUggacggucc............................................................................................................................................   3   1   LT-2
..........................................................................................uucuauggugaggacgguccg...........................................................................................................................................   1   0   JCK-2
```

Mature | Star

ucaccuaaauuauuuuggacggcaaguga uauaaaauugacacuuuuugauaaguuaguuuauauauauauauauauauauauacagaaauucuauggugaggacgguccguauaaggaccguaguauuagugacaauuuuucauaguauuaucgacgauuuuuuagaaaaucaucagcaauacuaugaaaaauuaucaucaauacugugauccguaugagaaccauccguaccauaaacggauugugua

uucuauggugaggacgguccg	1	0	LT-3
uucuauggugaUgacgguccg	2	1	LT-1
uucuauggugaggacgguccg	1	0	JCK-3
uucuauggugaggacgguccg	2	0	LT-2
uucuauggugaggacgguccg	1	0	JCK-1
uucuauggugaggacgguccAu	2	1	JCK-3
uucuauggugaggacgguccAu	2	1	LT-3
uucuauggugaggacgguccgu	1	0	LT-2
uucuauggugaggacgguccAu	3	1	JCK-2
uucuauggugaggacgguccAu	1	1	LT-1
uucuauggugaggacgguccguU	1	1	JCK-3
uucuauggugaggacgguccgua	1	0	LT-3
uucuauggugaggacgguccgNa	1	1	LT-2
uucuauggugaggacgguccguU	1	1	JCK-2
uucuauggugaggacgguccguau	3	0	LT-3
uucuaugCugaggacgguccguau	2	1	JCK-2
ucuauggugaggacggucc	2	0	LT-3
ucuaugguCaggacggucc	1	1	LT-3
ucuauggugaggacggucc	2	0	LCK-3
ucuauggugaggacgguccg	2	0	LT-3
ucuauggugaggacgguccgu	1	0	JCK-1
ucuauggugaggacgguccAu	1	1	JCK-3
ucuauggugaggacgguccAu	2	1	LT-2
ucuauggugaggacgguccAu	2	1	JCK-2
ucuauggugaggacgguccAu	1	1	LT-3
ucuauggugaggacgguccAu	1	1	LT-1
cuauggugaggacggucc	5	0	JT-3
cuauggugaggacggucc	5	0	JT-2
cuauggugaggacggucc	9	0	LCK-3
cuauggugaggacggucc	1	0	LT-3
uauggugaggacgguccgu	1	0	JCK-3
uauggugaggacgguccguau	3	0	LT-2
uauggugaggacgguccgCauaag	1	1	JCK-1
uauggugaggacgguccgCauaag	1	1	JCK-2
auggugaggacgguccgua	1	0	LCK-3
auggugaggacgguccguau	1	0	JCK-3
auggugaggacgguccguau	1	0	JCK-2
auggugaggacgguccgCauaagg	2	1	JCK-3
augAugaggacgguccguauaagg	1	1	LT-3
auggugaggacgguccgCauaagg	2	1	JCK-1
auAgugaggacgguccguauaagg	2	1	JCK-2
auAgugaggacgguccguauaagg	1	1	LT-1
auggugaggacggucUguauaagg	1	1	LT-1
auggugaggacgguccguauaagg	1	0	JCK-3
auggugaggacggucUguauaagg	1	1	LT-3
auAgugaggacgguccguauaagg	1	1	JCK-3
auggugaggacgguccgCauaagg	1	1	JCK-2
augAugaggacgguccguauaagg	1	1	LT-1
uggugaggacgguccgua	2	0	LT-2
ggugaggacgguccguaua	3	0	LT-2
gugaggacgguccguauaagg	1	0	LT-3
gugaggacgguccguauaagg	1	0	LT-1
ugaggacgguUcguauaagg	1	1	JCK-1
ugaggacgguUcguauaagg	3	1	JCK-2
ugaggacgguccguauaaggG	1	1	LT-1
ugaggacgguccguauaaggG	3	1	JCK-2
gaggacgguccAuauaag	1	1	LT-3
gaggacgguccguauaCgg	1	1	LT-1
gaggacgguccguauaagA	1	1	LT-3
gaggacgguccguauaagg	1	0	LT-3
gaggacgguccguauaagg	2	0	JCK-3
gaggacgguccguauaagg	2	0	LT-2
gaggacgguUcguauaagg	1	1	JCK-2
gaggacUguccguauaaggac	1	1	LT-3
ccguauaaggaccguaguauuagu	1	0	JCK-2
ccguauaaggaccgCaguauuagu	2	1	LT-1
ccgCauaaggaccguaguauuagu	1	1	JCK-3
cgCauaaggaccguaguauuagu	2	1	LT-3
cguauaaggaccguaguauuagu	1	0	LT-2
cguauaaggaccgCaguauuagu	1	1	JCK-3

Mature Star

ucaccuaaauuauuuuggacggcaagugauauaaaauugacacuuuuugauaaguuaguuuauauauauauauauauauauauacagaaauucuauggugaggacgguccguauaaggaccguaguauuagugacaauuuuucauaguauuaucgacgauuuuuuagaaaaucaucagcaauacuaugaaaaauuaucaucaauacugugauccguaugagaaccauccguaccauaaacggauugugua			
....cguauaaggaccgCaguauuagu..	1	1	JCK-1
....cgCauaaggaccguaguauuagug...	1	1	JCK-1
....guauaaggaccgCaguauuagug...	3	1	JCK-2
....gCauaaggaccguaguauuaguga..	1	1	JT-3
....gCauaaggaccguaguauuaguga..	1	1	JT-2
....gCauaaggaccguaguauuaguga..	3	1	JCK-2
....uauaaggaccgCaguauuagu..	1	1	LT-3
....uauaaggaccgCaguauuagu..	1	1	JCK-2
....Cauaaggaccguaguauuagu..	3	1	LT-2
....uauaaggaccgCaguauuagu..	1	1	LT-2
....uauaaggaccgCaguauuagugac...	1	1	LT-3
....uauaaggaccgCaguauuagugac...	1	1	JCK-1
....uauaaggaccgCaguauuagugac...	2	1	JCK-3
....uauaaggaccgCaguauuagugac...	2	1	JCK-2
....auaaggaccgCaguauuagug...	3	1	LT-1
....auaaggaccgCaguauuagug...	2	1	JCK-2
....auaaggaccguaguauuagug...	2	0	JCK-3
....auaaggaccgCaguauuagug...	1	1	JCK-1
....auaaggaccguaguauuagug...	1	0	JCK-2
....auaaggaccgCaguauuagug...	2	1	JCK-3
....auaaggaccgCaguauuagug...	4	1	LT-2
....auaagAaccguaguauuagugac...	1	1	JCK-2
....uaaggaccAuaguauuag...	1	1	LT-3
....uaaggaccguaguauuagug...	1	0	LT-1
....uaaggaccgCaguauuagug...	4	1	LT-2
....uaaggaccgCaguauuagug...	2	1	JCK-2
....uaaggaccgCaguauuagug...	4	1	LT-1
....uaaggaccgCaguauuagug...	1	1	LT-3
....uaaggaccguaguauuaguU...	1	1	LT-1
....uaaggaccgCaguauuagug...	3	1	LCK-3
....uaaggaccguaguauuaguU...	4	1	LCK-3
....uaaggaccgCaguauuagug...	1	1	JCK-1
....uaaggaccgCaguauuaguga..	2	1	LT-1
....uaaggaccgCaguauuaguga..	1	1	JCK-2
....uaaggaccguaguauuaguga..	1	0	JCK-1
....uaaggaccgCaguauuaguga..	3	1	JCK-3
....uaaggaccgCaguauuaguga..	6	1	LT-2
....uaaggaccguaguauuaguga..	2	0	JCK-2
....uaaggaccgCaguauuaguga..	6	1	LT-3
....uaaggaccgCaguauuaguga..	2	1	JCK-1
....uaaggaccguaguauuaguga..	1	0	JCK-3
....uaaggaccAuaguauuaguga..	1	1	LT-3
....uaaggaccgCaguauuaguga..	7	1	LCK-3
....uaagAaccguaguauuagugac...	3	1	JCK-2
....uaaggaccgCaguauuagugac...	2	1	LT-2
....uaaggaccgCaguauuagugac...	1	1	JCK-3
....uaagAaccguaguauuagugac...	1	1	JCK-3
....uaaggaccgCaguauuagugac...	1	1	LCK-2
....uaaggaccgCaguauuagugac...	2	1	LT-3
....uaaggaccgCaguauuagugaca..	1	1	LT-1
....aaggaccAuaguauuagug...	1	1	LT-1
....aaggaccguaguauuaguga..	6	0	JT-3
....aaggaccguaguauuaguga..	3	0	JT-2
....aaggaccgCaguauuaguga..	1	1	LT-3
....aaggaccgCaguauuagugac...	2	1	LT-1
....aaggaccgCaguauuagugac...	1	1	LT-2
....aaggaccgCaguauuagugac...	2	1	LT-3
....aaggaccguaguauuagugac...	1	0	LT-3
....aaggaccgCaguauuagugaca..	1	1	LT-3
....aaggaccgCaguauuagugaca..	5	1	LCK-3
....Gaggaccguaguauuagugaca..	1	1	LT-3
....aaggaccguaguauuagugacG..	2	1	LCK-3
....aaggaccguaguauuagugacGau..	3	1	JCK-3
....aaggaccguaguauuagugacGau..	1	1	JCK-1
....aaggaccguaguauuagugacaGu..	3	1	LT-1
....aaggaccguaguauuagugacGau..	4	1	JCK-2
....aggaccguaguauuagug...	1	0	JCK-3
....aggaccguaguauuaguga..	1	0	JCK-2
....aggaccguaguauuaguga..	1	0	LT-1
....aggaccguaguauuagugG..	1	1	JT-3

Mature ... Star

ucaccuaaauuauuuuggacggcaagugauauaaaauugacacuuuuugauaagguuaguuuauauauauauauauauauauauauacagaaauucuauggugaggacgguccguauaaggaccguaguauuagugacaauuuuucauaguauuaucgacgauuuuuuagaaaaucaucagcaauacuaugaaaaauuaucaucaauacugugauccguaugagaaccauccguaccauaaacggauugugua

aggaccguaguauuaguga	11	0	JT-3
aggaccguaguauuaguga	9	0	JT-2
aggaccguaguauuagugac	2	0	LT-3
agAaccguaguauuagugaca	1	1	LT-1
aggaccguaguauuagugaUa	2	1	LCK-3
aggaccguaguauuagugacGau	2	1	LT-3
aggaccguaguauuagugacaaA	1	1	LT-1
aggaccguaguauuagugacaGu	4	1	LCK-3
aggaccguaguauuagugacGau	1	1	LT-1
aggaccguaguauuagugacaGuu	1	1	LT-1
Agaccguaguauuagugaca	1	1	JCK-1
Agaccguaguauuagugaca	1	1	JCK-3
Agaccguaguauuagugaca	2	1	JCK-2
gaccguaguauuagugaca	1	0	LT-2
cguaguauuagugacaGuu	1	1	JCK-3
cguaguauuagugacaGuu	4	1	LT-2
uuagaaaaucGucagcaauacu	3	1	JCK-3
caucagcaauacuaugaaCa	9	1	LT-2
auuaucaucaauacugugauccgC	7	1	LCK-3
auuaucaucaauacugugauccgC	1	1	JCK-2
uaucaucaauacugugGuccgua	1	1	LT-1
caucaauacugugauccgC	1	1	JCK-3
caucaauacugugauccgC	2	1	JCK-2
caucaauacugugauccgCa	1	1	LT-1
caucaauacugugauccgCa	2	1	LT-2
caucaauacugugauccgCaug	1	1	JCK-3
caucaauacugugauccgCauga	1	1	LT-1
caucaauacugugauccgCauga	2	1	JCK-2
caucaauacugugauccgCaugag	4	1	JCK-3
caCcaauacugugauccguaugag	1	1	JCK-2
caucaauacugugauccgCaugag	2	1	JCK-2
caucaauacugugauccgCaugag	7	1	JCK-1
caCcaauacugugauccguaugag	3	1	LT-2
caucaauacugugauccgCaugag	1	1	LT-1
caCcaauacugugauccguaugag	1	1	JCK-1
aucaauacuguAauccgua	5	1	LT-2
aucaauacugugauccgCau	5	1	LCK-3
aucaauacugugauccgCaug	1	1	JCK-3
aucaauacugugauccgCaug	1	1	JCK-2
aucaauacugugauccgCauga	4	1	LT-2
aucaauacugugGuccguaugaga	1	1	LT-3
aucaauacugugGuccguaugaga	16	1	JCK-2
aucaauacugugGuccguaugaga	4	1	JCK-1
aucaauacugugGuccguaugaga	7	1	JCK-3
ucaauacugugauccgCaugag	1	1	LT-3
caauacuCugauccguaugaga	1	1	JCK-3
caauaUugugauccguaugaga	4	1	JCK-2
caauacugugGuccguaugaga	1	1	JCK-3
caauacugugGuccguaugaga	5	1	LCK-3
caauaUugugauccguaugaga	2	1	JCK-3
caauaUugugauccguaugaga	1	1	JCK-1
caauacugugGuccguaugagaa	2	1	LT-2
caauacugugGuccguaugagaac	4	1	LT-2
caauacugugauccguCugagaac	1	1	LT-1
caauacugugaucUguaugagaac	4	1	LCK-3
caauacugugaucUguaugagaac	1	1	LT-2
caauacugugauccguaugagaac	2	0	LT-3
aauacugugGuccguaugaga	1	1	JCK-3
aauacugugGuccguaugaga	3	1	LT-2
aauacugugGuccguaugagaa	1	1	JCK-2
aauacugugGuccguaugagaac	2	1	LT-1
aauacugugGuccguaugagaacc	3	1	JCK-1
aauacugugauccguaugCgaacc	1	1	JCK-2
aauacugugGuccguaugagaacc	5	1	JCK-3
aauacugugGuccguaugagaacc	1	1	LT-1
aauacuAugauccguaugagaacc	1	1	JCK-3
aauacuCugauccguaugagaacc	1	1	JCK-2
aauacuAugauccguaugagaacc	1	1	JCK-1
aauacugugGuccguaugagaacc	5	1	JCK-2
aauacugugauccguaugagaacc	2	0	JCK-2

```
                                                                                       Mature                                                                                                                                    Star
ucaccuaaauuauuuuggacggcaagugauauaaaauugacacuuuuugauaaguuaguuuauauauauauauauauauauauacagaaauucuauggugaggacgguccguauaaggaccguaguauuagugacaauuuuucauaguauuaucgacgauuuuuuagaaaaucaucagcaauacuaugaaaaauuaucaucaauacugugauccguaugagaaccauccguaccauaaacggauugugua
.........................................................................................................................................................................................................aauacuAugauccguaugagaacc.........................   1   1   JCK-2
.........................................................................................................................................................................................................Gauacugugauccguaugagaacca........................   2   1   LT-1
.........................................................................................................................................................................................................aauacugugGuccguaugagaacca........................   1   1   LT-3
..........................................................................................................................................................................................................auacugugGuccguaugaga............................   1   1   LT-3
..........................................................................................................................................................................................................auacugugGuccguaugagaac..........................   1   1   LT-1
..........................................................................................................................................................................................................auacugugGuccguaugagaac..........................   2   1   JCK-2
..........................................................................................................................................................................................................auacugugGuccguaugagaac..........................   3   1   JCK-1
..........................................................................................................................................................................................................auacugugGuccguaugagaac..........................   2   1   JCK-3
..........................................................................................................................................................................................................auacugugGuccguaugagaacc.........................   1   1   LT-3
..........................................................................................................................................................................................................auacuAugauccguaugagaacc.........................   2   1   JCK-2
..........................................................................................................................................................................................................auacugugGuccguaugagaacc.........................   1   1   LT-1
..........................................................................................................................................................................................................auacuAugauccguaugagaacc.........................   2   1   JCK-1
..........................................................................................................................................................................................................auacugugGuccguaugagaacc.........................   2   1   JCK-1
..........................................................................................................................................................................................................auacugugGuccguaugagaacc.........................   1   1   JCK-2
...........................................................................................................................................................................................................uacugugGuccguaugaga............................   2   1   LT-3
...........................................................................................................................................................................................................uacugugGuccguaugagaa...........................   1   1   JCK-2
...........................................................................................................................................................................................................uacugugGuccguaugagaa...........................   1   1   JCK-3
...........................................................................................................................................................................................................uacugugauccguaugagaa...........................   5   0   LCK-3
...........................................................................................................................................................................................................uacugugGuccguaugagaac..........................   1   1   LT-1
...........................................................................................................................................................................................................uacuAugauccguaugagaacca........................   1   1   LT-2
...........................................................................................................................................................................................................uacugugauccguaugagaaccG........................   1   1   JCK-2
...........................................................................................................................................................................................................uacugugauccguaugagaaccaA.......................   19  1   LCK-1
...........................................................................................................................................................................................................uacugugauccguaugagaaccaA.......................   16  1   LCK-2
............................................................................................................................................................................................................acugugauccguaugagaac..........................   2   0   LT-1
............................................................................................................................................................................................................acugugauccguaugagGacc.........................   1   1   LT-1
............................................................................................................................................................................................................acugugauccguaugagaacc.........................   1   0   LT-3
............................................................................................................................................................................................................acugugauccgCaugagaaccauc......................   1   1   LT-2
............................................................................................................................................................................................................acugugauccguaugagaaccauc......................   14  0   LT-2
............................................................................................................................................................................................................acugugauccguaugagaaccauc......................   4   0   LT-3
............................................................................................................................................................................................................acugugauccguaugagaaccauc......................   22  0   LT-1
............................................................................................................................................................................................................acugugauccgCaugagaaccauc......................   4   1   LT-3
............................................................................................................................................................................................................acugugauccguaugaAaaccauc......................   3   1   LT-1
............................................................................................................................................................................................................acuAugauccguaugagaaccauc......................   2   1   LT-1
............................................................................................................................................................................................................acugugauccguaugagaaccauc......................   2   0   JCK-1
............................................................................................................................................................................................................acugugauccgCaugagaaccauc......................   1   1   LT-1
............................................................................................................................................................................................................acugugauccguaugagaaccGuc......................   6   1   LCK-3
............................................................................................................................................................................................................acugugaucUguaugagaaccauc......................   1   1   LT-3
............................................................................................................................................................................................................acugugauccgCaugagaaccauc......................   3   1   JCK-3
............................................................................................................................................................................................................acugugauccguaugagaaccauc......................   7   0   JCK-2
............................................................................................................................................................................................................Ccugugauccguaugagaaccauc......................   1   1   LT-2
............................................................................................................................................................................................................acugugauccguaugaAaaccauc......................   1   1   LT-2
............................................................................................................................................................................................................acugugauccguCugagaaccauc......................   1   1   LT-3
............................................................................................................................................................................................................acugugauccgCaugagaaccauc......................   5   1   LCK-3
............................................................................................................................................................................................................acugugauccguaugagaaccaucA.....................   3   1   LT-1
.............................................................................................................................................................................................................cugugauccguaugagaac..........................   4   0   LT-3
.............................................................................................................................................................................................................cugugGuccguaugagaac..........................   2   1   LT-3
.............................................................................................................................................................................................................cuAugauccguaugagaac..........................   5   1   LCK-3
.............................................................................................................................................................................................................cugugauccguaugagaac..........................   4   0   LT-2
.............................................................................................................................................................................................................cuAugauccguaugagaacc.........................   6   1   LCK-3
.............................................................................................................................................................................................................cugugauccguaugagaaccG........................   2   1   JCK-2
.............................................................................................................................................................................................................cugugauccguaugagaaccG........................   1   1   JCK-3
.............................................................................................................................................................................................................cugugauccguaugagaaccG........................   1   1   JCK-1
.............................................................................................................................................................................................................cugugauccguaugagaaccGuc......................   4   1   LCK-3
.............................................................................................................................................................................................................cugugauccguaugagaaccGucc.....................   4   1   JCK-3
.............................................................................................................................................................................................................cugugauccguaugagaaccGucc.....................   2   1   JCK-1
.............................................................................................................................................................................................................cugugauccguaugagaaccaucc.....................   1   0   JCK-2
.............................................................................................................................................................................................................cugugauccguaugagaaccGucc.....................   2   1   JCK-2
..............................................................................................................................................................................................................ugugauccguaugagaacc.........................   2   0   LT-3
..............................................................................................................................................................................................................uAugauccguaugagaacc.........................   3   1   LT-2
..............................................................................................................................................................................................................ugugauccguaugagaaccGucc.....................   1   1   LT-2
...............................................................................................................................................................................................................Augauccguaugagaaccaucc.....................   1   1   LT-3
...............................................................................................................................................................................................................Cugauccguaugagaaccaucc.....................   1   1   LT-3
...............................................................................................................................................................................................................gugauccguaugagaaccauccgC...................   2   1   LT-3
....................................................................................................................................................................................................................ccguaugagaaccGuccgua..................   3   1   LT-2
...............................................................................................................................................................................................................................ccauccguaccauaGacg.........   2   1   LT-1
```
